# A CRISPR/Cas12a-based reverse transcription multiple cross displacement amplification technique for sensitive, rapid, and highly specific detection of *human enterovirus A71* in clinical application

**DOI:** 10.3389/fmicb.2026.1883920

**Published:** 2026-06-19

**Authors:** Xujian Zhang, Rui Ye, Qi Liang, Yumei Cao, Mao Liu, Fang Wei, Yonglin Zhu, Yan Yuan, Guo Guo, Yu Wang

**Affiliations:** 1 School of Basic Medical Sciences, Guizhou Key Laboratory of Microbio and Infectious Disease Prevention and Control, Guizhou Medical University, Guiyang, Guizhou, China; 2 Department of Clinical Laboratory, The First People's Hospital of Guiyang, Guiyang, Guizhou, China; 3 Department of Basic Clinical Laboratory Medicine, School of Clinical Laboratory Science, Guizhou Medical University, Guiyang, Guizhou, China; 4 School of Public Health, Key Laboratory of Environmental Pollution Monitoring and Disease Control, Ministry of Education, Guizhou Medical University, Guiyang, Guizhou, China

**Keywords:** CRISPR/Cas12a system, *enterovirus A71*, multiple cross displacement amplification, rapid diagnostic method, VP1 gene

## Abstract

*Enterovirus A71* (EVA71) is a major pathogen responsible for hand, foot, and mouth disease (HFMD) outbreaks and poses a considerable threat to public health. In this work, we established a molecular detection system that integrates reverse transcription multiple cross displacement amplification (RT-MCDA) with CRISPR/Cas12a technology, targeting the VP1 gene region for EVA71 identification. This platform, termed EVA71-RT-MCDA-CRISPR, utilizes RT-MCDA to achieve efficient pre-amplification of the target sequence, followed by sequence-specific recognition mediated by CRISPR/Cas12a. The detection results can be observed both through real-time fluorescence analysis and visual inspection under blue light illumination. The assay exhibits outstanding sensitivity, achieving a limit of detection as low as 3.36 copies per reaction, while retaining high specificity against non-EVA71 templates. Validation using clinical specimens further confirmed the diagnostic reliability of this RT-MCDA-CRISPR platform. The successful establishment of the EVA71-RT-MCDA-CRISPR system demonstrates its potential as a rapid, user-friendly, highly sensitive and specific method for on-site EVA71 detection, serving as a valuable tool for clinical diagnosis and epidemiological surveillance.

## 1 Introduction

*Enterovirus* A71 (EVA71), classified within the *Picornaviridae* family as a member of the *Enterovirus A* species, is a non-enveloped RNA virus (Zhu et al., 2023). Initially identified in 1969 from a pediatric case presenting with central nervous system disorders in California, this pathogen has since been extensively studied (Wang et al., 2022). Recognized as a primary etiological agent of hand, foot, and mouth disease (HFMD), EVA71 infections can, in certain cases, progress to severe neurological complications, including acute flaccid myelitis (AFM) (Zhang W. et al., 2024). The mechanisms underlying EVA71 pathogenesis remain incompletely understood (Chen and Ling, 2019). However, emerging evidence suggests a direct correlation between the severity of HFMD symptoms and the quantity of viral genetic material detected in samples (Chen and Ling, 2019; Wei et al., 2024). Structurally, the EVA71 genome is enclosed within an icosahedral capsid composed of 60 repeating units of the structural proteins VP1 through VP4(Xie et al., 2024). Among these, VP1 serves as the principal antigenic determinant and plays a critical role in viral pathogenicity. Mutations in the VP1 protein have been reported to significantly influence the virulence of EVA71 (Xu and Li, 2024). Widespread vaccination with EVA71 inactivated vaccines has greatly lowered the incidence of EVA71-related outbreaks (Bifani et al., 2025). *Coxsackievirus* A16 (CVA16), A10 and A6 have become the predominant circulating strains, which usually only induce mild symptoms (Liu et al., 2025). In contrast, EVA71 remains the major pathogen causing severe cases and deaths (Guan et al., 2017; Liu et al., 2025). Furthermore, the virus possesses strong environmental tolerance and is prone to genetic mutation (Kitamura and Arita, 2024; Wang S. et al., 2024). Therefore, developing rapid and accurate diagnostic methods for EVA71 is essential. Such assays can distinguish EVA71 from other common enteroviruses and provide support for early warning, clinical triage and targeted prevention and control of EVA71 infection.

The conventional method for detecting EVA71 involves viral cultivation in cell culture; however, this approach is labor-intensive and time-consuming (Puenpa et al., 2019). Although VP1 gene sequencing is widely recognized as the reference standard for genetic classification and is recommended by WHO guidelines for *enterovirus* surveillance, its high cost restricts broad implementation (Kitamura and Arita, 2024; Yeh-Sheng et al., 2023). Advanced molecular diagnostics, including RT-qPCR, demand specialized instruments, costly reagents, and well-established nucleic acid extraction protocols. When coupled with the need for skilled operators, these requirements render them unsuitable for urgent clinical or emergency diagnostic settings (Chen et al., 2023; Coutinho et al., 2020). These limitations highlight the urgent need to improve detection methodologies, creating a strong demand for diagnostic approaches that are simple, rapid, highly sensitive, specific, and accurate. Such developments would support timely therapeutic interventions and more effective control of EVA71 outbreaks.

Multiple cross displacement amplification (MCDA) is a isothermal nucleic acid amplification method capable of rapid, highly specific, and sensitive detection of DNA/RNA under constant temperature conditions (Wang et al., 2016). Overcoming the drawbacks of conventional methods, MCDA only requires basic temperature-control equipment, which greatly reduces instrument dependence, making it well-suited for point-of-care testing (POCT) with results obtainable within 40 min (Xiao et al., 2024). It has been successfully applied to the detection of various pathogenic microorganisms, including bacteria (Huang et al., 2023), viruses (Sun et al., 2022), and fungi (Jia et al., 2025a). Nevertheless, end-point detection techniques for MCDA amplicons such as gel electrophoresis, turbidity measurement, and colorimetric assays often yield false-positive outcomes caused by nonspecific amplification (Mao et al., 2025; Zhang et al., 2025). To address these shortcomings, the CRISPR/Cas (clustered regularly interspaced short palindromic repeat-associated protein) system has been introduced as an advanced molecular detection platform. It is renowned for its capability to perform highly precise sequence recognition and produce unambiguous diagnostic readouts (Jia et al., 2025b). The CRISPR/Cas-based detection mechanism relies on two key processes: precise identification of nucleic acid targets and the collateral cleavage activity of Cas enzymes such as Cas12, Cas13, and Cas14(Jia et al., 2025b, c). Upon recognition of the target DNA and its associated protospacer adjacent motif (PAM) site under the direction of a guide RNA (gRNA), Cas12a (previously designated as Cpf1) initiates non-specific collateral cleavage of adjacent fluorophore-tagged single-stranded DNA (ssDNA)(Pickar-Oliver and Gersbach, 2019). This process generates a measurable fluorescent readout. A key advantage of this design is its ability to prevent false-positive results typically caused by non-specific products from isothermal amplification (Jia et al., 2023; Wang X. et al., 2024; Zhu et al., 2021).

**Figure 1:**
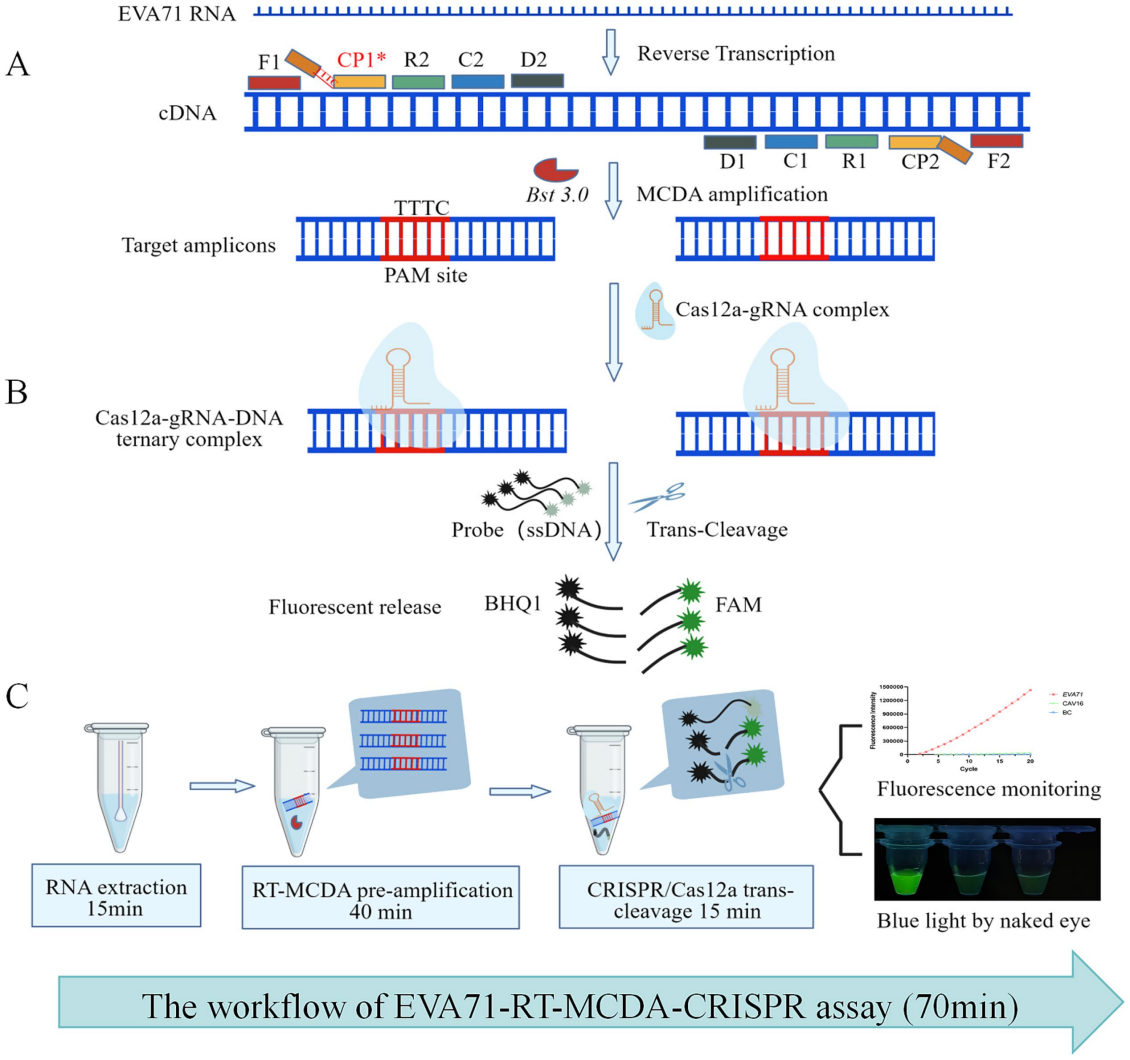
Schematic illustration of the principle of the EVA71-RT-MCDA-CRISPR assay. **(A)** EVA71 RNA was reverse-transcribed into cDNA first. In the RT-MCDA reaction, CP1* primers with PAM sequence (TTTC) modified in the linker region were used to amplify specific products containing PAM sites. **(B)** After the Cas12a-gRNA complex specifically recognizes the target DNA, Cas12a exhibits activated trans-cleavage activity to non-specifically cleave 5’-FAM and 3’-BHQ1 dual-labeled ssDNA reporter probes, releasing fluorescent signals. **(C)** Schematic workflow of the EVA71-RT-MCDA-CRISPR assay. The entire detection system consists of four steps: RNA extraction (15 min), RT-MCDA pre-amplification (40 min), CRISPR/Cas12a trans-cleavage with fluorescence monitoring (15 min), and the total procedure can be completed within 70 min.

In this context, we developed an innovative diagnostic approach by integrating CRISPR/Cas12a technology with RT-MCDA amplification, resulting in a system capable of rapid, precise, ultra-sensitive, and highly specific identification of EVA71 infections. The developed EVA71-RT-MCDA-CRISPR assay demonstrates excellent stability and reproducibility, providing a robust solution for EVA71 detection. In addition, the operational mechanism of the EVA71-RT-MCDA-CRISPR detection system is clearly illustrated (Figure 1), and its practical applicability was validated through testing with clinical samples.

## 2 Materials and methods

### 2.1 Reagents and instruments

The following reagents and equipment were sourced from various suppliers: New England BioLabs (USA) provided the 10 × isothermal amplification buffer II, magnesium sulfate solution, *Bst 3.0* DNA Polymerase (Product Code: M0374L), deoxynucleotide (dNTP) mixture (Product Code: N0447L), EnGen^®^ Lba Cas12a (Cpf1) enzyme, and 10 × NEBuffer™ r2.1 (Product Code: M0653T). Xi’an Tianlong Technology Co., Ltd. (Xi’an, China) supplied the nucleic acid extraction kits (Product Code: qEX-DNA/RNA Virus 4.0). Vazyme Biotech Co., Ltd. (Nanjing, China) provided the T7 High Yield RNA Transcription Kit (Product Code: TR101). The Applied Biosystems 7,500 Fast Real-Time PCR System was obtained from Applied Biosystems Inc. (USA). Chuanghe Biotech Co., Ltd. (Jiangsu, China) manufactured the quantitative PCR detection kits (Product Code: CN08-4A) for *Enterovirus* A71 identification. For spectrophotometric measurements, the WD-2112A ultra-micro model was sourced from Beijing Liuyi Biotechnology Company (China), while the E-Box CX5 gel documentation system was procured from Vilber Lourmat (France).

### 2.2 Primers and gRNA design

Five primer pairs targeting the conserved VP1 region of EVA71 (GenBank accession: GQ279370.1) were designed using PREMIER 5.0 software, and their specificity was subsequently confirmed via BLAST analysis. The complete MCDA primer set consists of two displacement primers (F1 and F2), six amplification primers (C1, C2, R1, R2, D1, D2), and two cross primers (CP1 and CP2). In the CRISPR/Cas12a system, the CP1* primer was specifically engineered to carry a Cas12a-recognizable sequence (TTTC). Additionally, a ssDNA reporter probe was synthesized, bearing a FAM fluorophore at its 5′ end and a BHQ1 quencher at the 3′ end. Figure 1 and Supplementary Figure S1 illustrate the spatial arrangements, while Table 1 lists the nucleotide sequences of all primers, guide RNA, and the detection probe.

**Table 1:** Sequences and modification in this study.

Primer/plasmid name^a^	Sequences and modifications	Length^b^	Gene
F1	5’-GCAGGTTTCAGTGCCATT-3’	18 nt	VP1
F2	5’-CATCGGGCGAGGTATCC-3’	17 nt	
CP1*	5'-CTTTCTCCTGTTTGTGTTCTCCGAA-**TTTC**-TGCGAGTGCTTATCAATGG-3'	48 mer	
CP2	5’-TCTCAGTGCGGACTGTGGGGTGACGTGCTTCATTCTCATG-3’	40 mer	
C1	5’-CTTTCTCCTGTTTGTGTTCTCCGAA-3’	25 nt	
C2	5’-TCTCAGTGCGGACTGTGGGG-3’	20 nt	
D1	5’-TGTGGGATATCCGTCATA-3’	18 nt	
D2	5’-AGTACCCTTTAGTGGTTAG-3’	19 nt	
R1	5’-CATGCCCCATATTCAAGAT-3’	19 nt	
R2	5’-TCCTAATAACATGATGGGCA-3’	20 nt	
gRNA	UAAUUUCUACUAAGUGUAGAUCUGCGAGUGCUUAUCAAUGG	41 nt	
Probe	FAM-TATTATTATTATTATTT-BHQ1	17 nt	
The cloned part of VP1 coding sequence (EVA71-VP1 plasmid)	GTCAAGCTGTCAGACCCTCCATCGCAGGTTTCAGTGCCATTCATGTCACCTGCGAGTGCTTATCAATGGTTTTATGACGGATATCCCACATTCGGAGAACACAAACAGGAGAAAGATCTTGAATATGGGGCATGTCCTAATAACATGATGGGCACGTTCTCAGTGCGGACTGTGGGGACCTCCAAGTCCAAGTACCCTTTAGTGGTTAGGATTTACATGAGAATGAAGCACGTCAGGGCGTGGATACCTCGCCCGATGCGTAACCAGAACT		

^a^CP1*The bolded nucleotide sequence TTTC within this primer is a genetically engineered inserted PAM site required for the EVA71-RT-MCDA-CRISPR detection system. ^b^ mer: monomeric; nt: nucleotide.

### 2.3 RNA standard construction

To generate EVA71 RNA standards, complete genomic sequences of EVA71 were retrieved from the NCBI GenBank database. These sequences underwent comparative alignment using MEGA11 software to identify highly conserved regions. A 271-bp fragment located within the VP1 gene was then selected and cloned into a pGEMT Easy vector that carries a T7 promoter sequence, thereby constructing a recombinant plasmid. This cloning procedure was performed by Beijing Tianyi Huiyuan Biotechnology Co., Ltd. (Beijing, China). The resulting plasmid served as the template for *in vitro* transcription (IVT). RNA standards were synthesized and purified using the T7 High Yield Transcription Kit (Vazyme Biotech Co., Ltd., Nanjing, China) in combination with the RNA Clean & Concentrator-5 Kit (Zymo Research, USA).

The EVA71 RNA concentration was determined to be 5.14 ± 0.1 ng/μL by measuring absorbance at 260/280 nm using WD-2112A ultra-micro spectrophotometer. The copy of RNA standard was calculated using the following formula: RNA copies/mL = [RNA concentration (ng/ mL) / (nt transcript length×340)] × 6.022 × 10^23^(Zhang X. et al., 2024).

### 2.4 EVA71-RT-MCDA pre-amplification reaction

RT-MCDA pre-amplification was conducted in a 25 μL reaction system. The final mixture contained displacement primers F1 and F2 (0.4 μM each), amplification primers C1, C2, R1, R2, D1, and D2 (0.8 μM each), and cross primers CP1 and CP2 (1.6 μM each). To this were added 2.5 μL of 10 × isothermal buffer II, 1.5 μL of 6 mM MgSO_4_, 3.5 μL of 1.4 mM dNTP mix, 1 μL of *Bst 3.0* DNA polymerase (8 U), and 1 μL of AMV reverse transcriptase (10 U). Template input was set at 1.0 μL for EVA71 RNA samples and 5.0 μL for clinical specimens. The volume was then adjusted to 25 μL with RNase-free water.

As validated in previous studies (Cheng et al., 2024), the RT-MCDA reaction was carried out at 65 °C for 40 min to ensure optimal amplification conditions.

### 2.5 CRISPR/Cas12a trans-cleavage assay

The CRISPR/Cas12a system was employed to detect MCDA-amplified products through a two-step procedure. In the initial step, the Cas12a-gRNA complex was assembled through the combination of 10 μL of Cas12a enzyme (1 μM) with an equivalent volume of gRNA (1 μM) in 80 μL of 2 × NEBuffer, followed by an incubation period at 37 °C for 8–10 min. This preformed complex could be used immediately or stored at 0–4 °C for up to 12 h (Jia et al., 2023; Zhu et al., 2021). In the second step, the CRISPR/Cas12a detection reaction was carried out in a 50 μL mixture consisting of 25 μL of 2 × NEBuffer, 9 μL of the pre-assembled Cas12a-gRNA complex, 1 μL of RT-MCDA amplification products, 1.25 μL of ssDNA reporter, and 13.75 μL of RNase-free water. The cleavage activity of CRISPR/Cas12a was measured at 35–40 °C for 5–20 min using an ABI 7500 Real-Time PCR instrument. During the reaction, real-time fluorescence signals were continuously recorded by the quantitative PCR instrument, and end-point results could also be visually inspected under blue light illumination. To avoid cross-contamination, filter tips were used throughout the experiment, reagent mixing and template addition were carried out in separate physical spaces. All operations were performed inside a Class II biological safety cabinet.

### 2.6 Optimization of CRISPR-Cas12a-mediated reaction

To identify the optimal reaction conditions for the CRISPR/Cas12a detection phase, a series of optimization experiments were carried out. We tested the reaction temperature at six different values (35 °C, 36 °C, 37 °C, 38 °C, 39 °C, and 40 °C) while maintaining the reaction time at 20 min. Next, reaction times ranging from 5 to 20 min were evaluated at 5 min intervals. After that, the Cas12a-gRNA complex volume was optimized across a range of 5 to 10 μL in 1 μL steps. Then, the fluorescent ssDNA reporter volume was optimized using volumes from 0.8 to 1.3 μL in 0.1 μL increments. Lastly, the RT-MCDA template volume added to the detection system was tested over a range of 0.5 to 3 μL in 0.5 μL steps. Each experimental condition was independently repeated three times.

### 2.7 Sensitivity, specificity and reproducibility of the EVA71-RT-MCDA-CRISPR assay

To determine the detection limit of the EVA71-RT-MCDA-CRISPR assay, EVA71 RNA was serially diluted to final concentrations of 3.36 × 10^7^, 3.36 × 10^6^, 3.36 × 10^5^, 3.36 × 10^4^, 3.36 × 10^3^, 3.36 × 10^2^, 3.36 × 10^1^, 3.36 × 10^0^, and 3.36 × 10^−1^ copies/μL. Each dilution was used as a template and tested in three independent replicates. Subsequently, the minimum detectable concentration was determined based on the positive detection rate among these replicates. For specificity assessment under optimized reaction conditions, eight different EVA71 isolates along with sixteen non-EVA71 microbial pathogens were subjected to the assay.

To evaluate operator variability, two operators performed the assay on EVA71 RNA at low (3.36 × 10^0^ copies/μL) and high (3.36 × 10^6^ copies/μL) concentrations, with three replicates for each concentration. Regarding Intra-batch reproducibility, EVA71 RNA at three concentration levels (3.36 × 10^0^, 3.36 × 10^3^, and 3.36 × 10^6^ copies/μL, corresponding to low, medium, and high concentrations respectively) was tested. Each concentration was replicated three times within a single run. For inter-batch reproducibility, samples of the same concentrations were detected in three independent batches, and the coefficient of variation (CV) was calculated accordingly. The formula for the coefficient of variation is as follows: Coefficient of CV was calculated as CV = (standard deviation/mean) × 100% (Chen et al., 2026; Qi et al., 2026).

### 2.8 Clinical feasibility verification of the EVA71-RT-MCDA-CRISPR detection system

To evaluate the clinical applicability of the EVA71-RT-MCDA-CRISPR assay, a retrospective analysis was conducted on 96 clinical specimens collected from patients with suspected HFMD at The First People’s Hospital of Guiyang. These specimens included 44 anal swabs and 52 throat swabs. All swabs were collected using sterile flocked devices. For anal sampling, the swab was gently rotated along the perianal folds and anal canal wall for 5–10 s. For throat sampling, the bilateral tonsils and posterior pharyngeal wall were swabbed three times, avoiding contact with the tongue or buccal mucosa. After collection, each swab was returned to its sterile transport sleeve and sealed. Specimens were transported at 2–8 °C; those not processed immediately were stored at −80 °C. Upon receipt, 1–2 mL of sterile saline was added to each tube, followed by vigorous vortex mixing. A 200 μL aliquot of the suspension was then used for nucleic acid extraction using a commercial viral RNA extraction kit (Tianlong Technology, Xi’an, China) on an automated magnetic-bead system, with a final elution volume of 60 μL. Each sample was analyzed in parallel using both the developed EVA71-RT-MCDA-CRISPR method and conventional RT-qPCR. The study was approved by the Ethics Committee of the First People’s Hospital of Guiyang City (Approval No. G2024-S022). Written informed consent was obtained from all participants in accordance with the Declaration of Helsinki.

## 3 Results

### 3.1 Operational principles of the EVA71-RT-MCDA-CRISPR diagnostic platform

The EVA71-RT-MCDA-CRISPR assay for detecting EVA71 follows a two-step workflow. In the first step, the target sequence is pre-amplified via RT-MCDA, and in the second step, the amplified products are recognized by a CRISPR/Cas12a-mediated trans-cleavage system (Figure 1). During the amplification phase, the target gene is subjected to isothermal amplification for 40 min using a set of ten primers. Notably, the cross primer CP1* contains a Cas12a-specific PAM sequence (TTTC) within its linker region, which facilitates efficient recognition by the Cas12a protein. As a result, abundant amplicons carrying the PAM site are produced (Figure 1A). In the detection phase, the CRISPR/Cas12a-gRNA complex specifically identifies the PAM site and binds to the target sequence under the direction of gRNA. This binding induces the formation of a ternary complex and activates the trans-cleavage activity of Cas12a. The activated Cas12a then indiscriminately cleaves the ssDNA reporter probe, generating a detectable fluorescent signal (Figure 1B). Fluorescence can be quantified using a real-time PCR instrument or visually assessed under blue light illumination. The entire procedure from sample to result can be completed within 70 min (Figure 1C).

### 3.2 Verification of the EVA71-RT-MCDA-CRISPR assay

**Figure 2:**
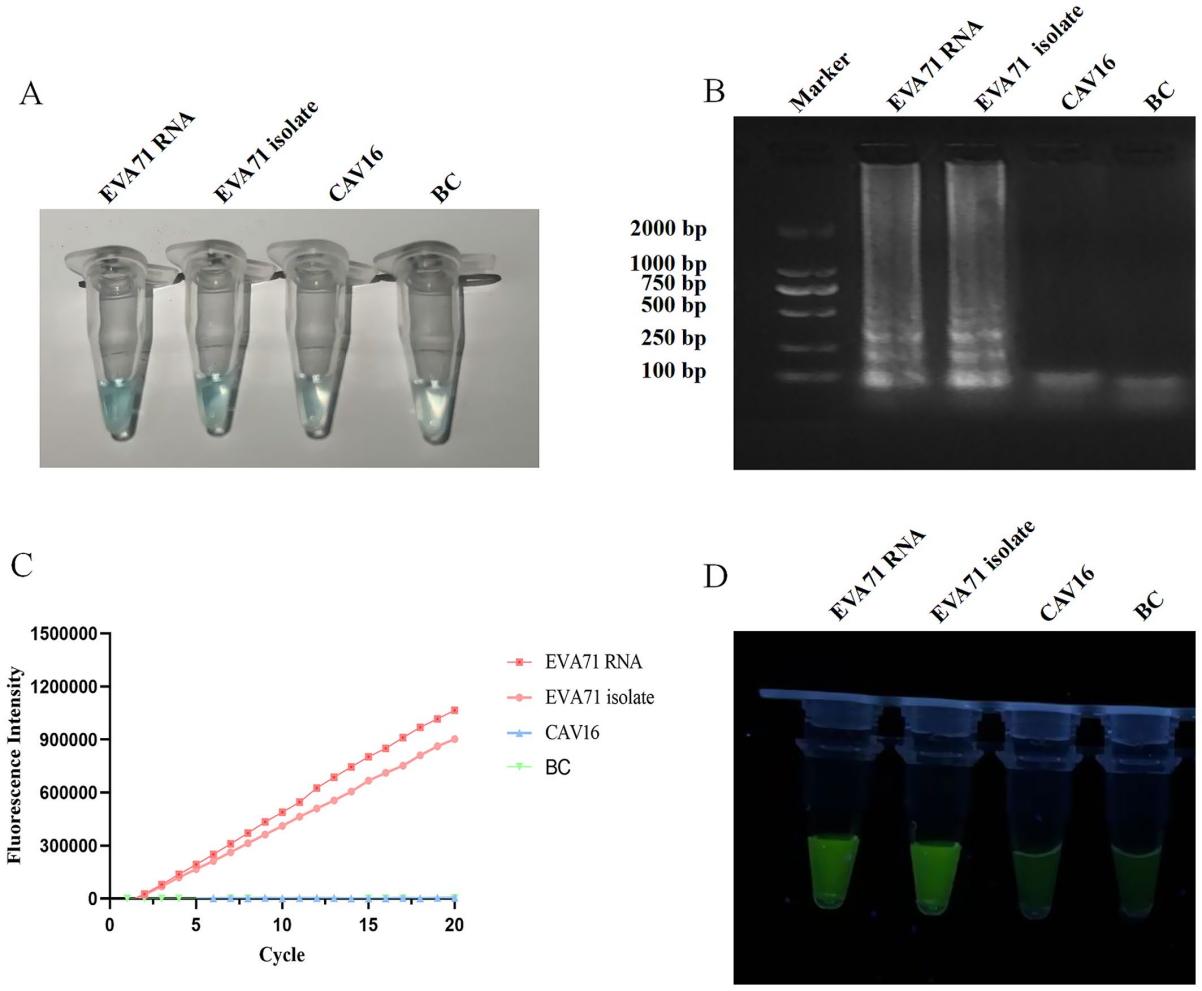
Verification of the EVA71-RT-MCDA-CRISPR assay. **(A)** The VDR method and **(B)** Agarose gel electrophoresis were employed for the EVA71 RT-MCDA pre-amplification products. **(C)** The target amplicons were cleaved by the CRISPR/Cas12a system, and the fluorescence signals were monitored via a real-time PCR system. **(D)** Visual observation was conducted under blue light. The EVA71 RNA standard and EVA71 isolate RNA were used as positive controls, the virus RNA of CVA16 was used as a negative control, and RNase–free water served as the blank control (BC).

The validation procedure comprised separate assessments of RT-MCDA pre-amplification products and CRISPR/Cas12a cleavage products. Positive controls consisted of EVA71 RNA standard and EVA71 isolate RNA, while negative controls used CVA16 RNA, with RNase-free water serving as blank controls. After 40 min of RT-MCDA pre-amplification at 65 °C, the resulting amplicons were evaluated using two methods: visual detection reagent (VDR) assays and 2% agarose gel electrophoresis. In the VDR analysis, a light green color appeared exclusively in reactions containing EVA71 RNA and EVA71 isolate RNA (Figure 2A). Simultaneously, gel electrophoresis exhibited the characteristic ladder-like banding pattern of MCDA products (Figure 2B). Subsequent CRISPR/Cas12a-based validation demonstrated that strong fluorescence signals were generated only from amplification products derived from EVA71 RNA and EVA71 isolate RNA (Figure 2C). Moreover, under blue-light illumination, strong fluorescent signals were exclusively observed in the positive samples, while no such signals were identified in the negative or blank controls (Figure 2D). Collectively, these results validate the feasibility and reliability of the EVA71-RT-MCDA-CRISPR system for the detection of EVA71.

### 3.3 Optimization of reaction conditions for EVA71-RT-MCDA-CRISPR assay

Previous studies have demonstrated that 65 °C is the optimal temperature for the pre-amplification stage of EVA71 detection using the RT-MCDA assay (Supplementary Figure S2; Cheng et al., 2024). In this study, we sought to identify the optimal reaction conditions for the subsequent CRISPR/Cas12a detection phase. A series of experiments were carried out to assess the system performance within a temperature gradient ranging from 35 °C to 40 °C. It was discovered that the optimal temperature for the CRISPR/Cas12a detection step was 37 °C, since this condition produced the most rapid and intense signal output (Supplementary Figure S3). To determine the ideal reaction duration for Cas12a trans-cleavage activity, we performed incubations at 37 °C for 5, 10, 15, and 20 min. Our results showed that the ssDNA reporter probe was efficiently cleaved within 5 min, while the maximum fluorescence signal was achieved at 15 min (Supplementary Figure S4). Based on these observations, we established 15 min at 37 °C as the standard reaction condition for the EVA71-RT-MCDA-CRISPR detection system, and this protocol was consistently applied in all subsequent experiments.

**Figure 3:**
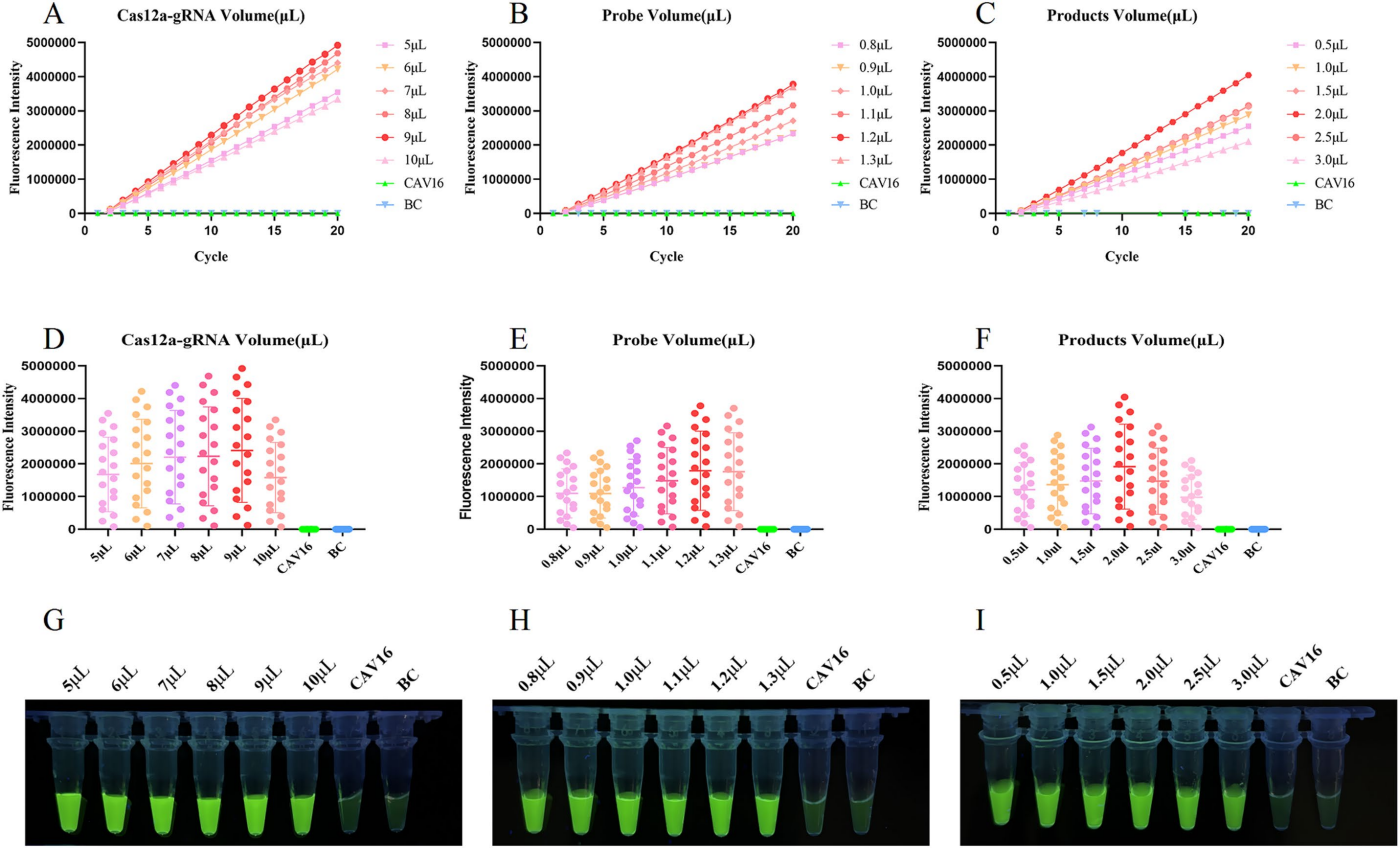
Optimization of the conditions for the EVA71-RT-MCDA-CRISPR assay. **(A)** Results of varying volumes of the Cas12a-gRNA complex. **(B)** Effects of varying volumes of the ssDNA reporter probe. **(C)** Varying volumes of MCDA pre-amplified products. **(D–F)** Demonstration of differential fluorescence intensity corresponding to varying volumes of the Cas12a-gRNA complex, the probe, and the products in the EVA71-RT-MCDA-CRISPR assay. **(G–I)** Results of varying volumes of the Cas12a-gRNA complex, the probe, and the products in the EVA71-RT-MCDA-CRISPR assay under blue–light illumination.

In parallel, we systematically optimized the working concentrations of three key components within the CRISPR/Cas12a reaction mixture: the Cas12a-gRNA complex, the ssDNA reporter probe, and the MCDA pre-amplified products. These adjustments were intended to enhance the efficiency of Cas12a-mediated nucleic acid cleavage. For the Cas12a-gRNA complex, tested volumes ranged from 5 to 10 μL, and subsequent analysis identified 9 μL as the most effective working volume (Figures 3A,D,G). The ssDNA reporter probe was examined across a concentration range of 0.8–1.3 μL, with optimal performance observed at 1.2 μL (Figures 3B,E,H). The MCDA pre-amplified products were evaluated at volumes between 0.5 and 3 μL, and the experimental data indicated that 2 μL was the ideal volume (Figures 3C,F,I).

### 3.4 Sensitivity of the EVA71-RT-MCDA-CRISPR assay

**Figure 4:**
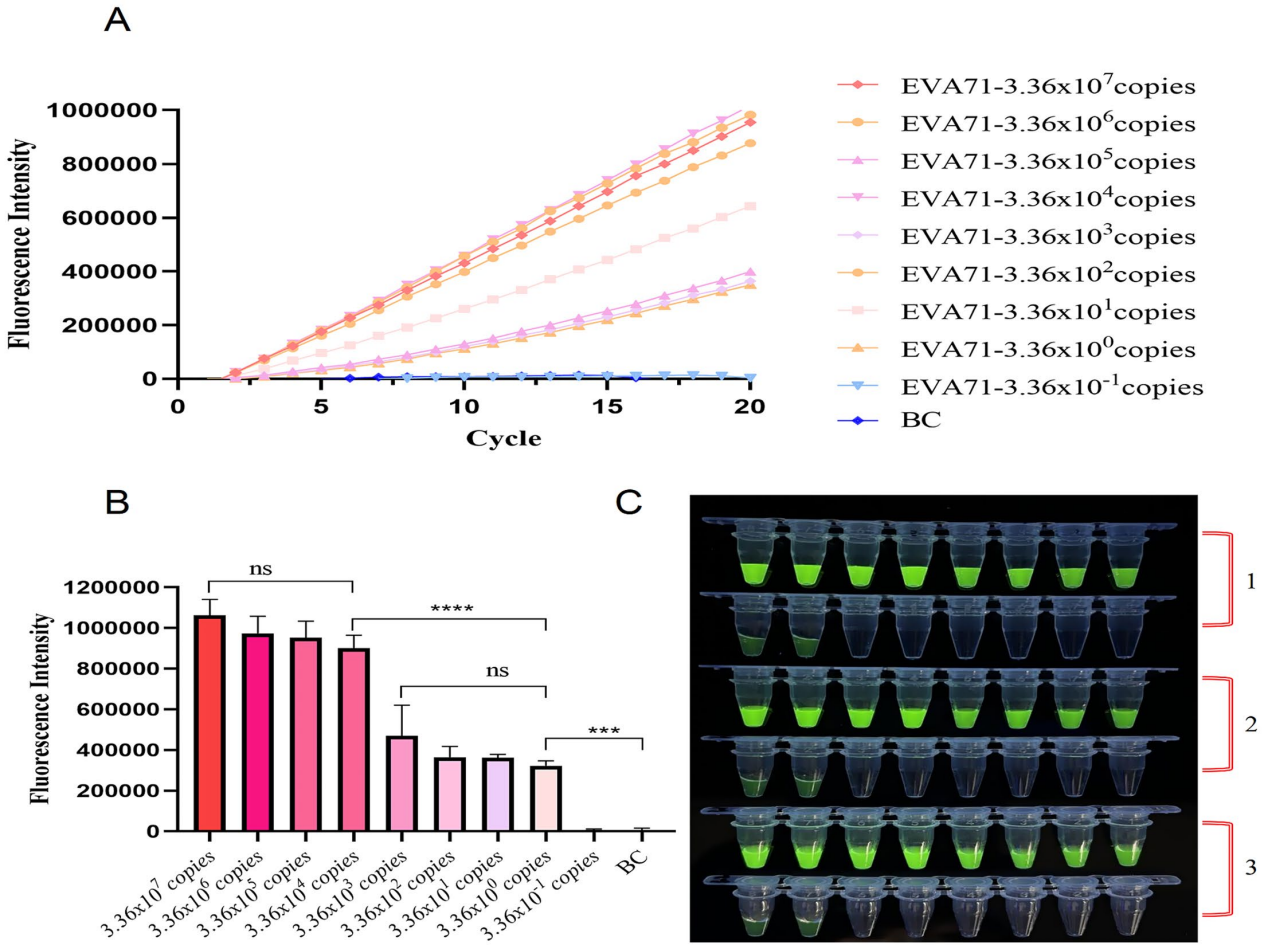
Sensitivity evaluation of the EVA71-RT-MCDA-CRISPR assay. Analytical sensitivity was assessed using serially diluted EVA71 RNA templates. **(A)** The fluorescence signals of serially diluted EVA71 RNA samples were recorded using a real-time fluorescence detector. **(B)** A two-tailed Student’s t test was conducted. Error bars represented mean ±S.E.M. (*n* = 3). S.E.M., standard error of mean. **** *p* < 0.0001; *** *p* < 0.001; ns, no significant. **(C)** It was visualized with the naked eye under blue light. Each assay was carried out in triplicate to guarantee reproducibility.

To evaluate the detection sensitivity of the EVA71-RT-MCDA-CRISPR method, a series of tenfold serial dilutions of EVA71 RNA templates, spanning concentrations from 3.36 × 10^7^ to 3.36 × 10^−1^ copies/μL, were tested. The experiments were conducted under pre-optimized reaction conditions, and the results were interpreted using both real-time fluorescence monitoring and visual inspection under blue light illumination. Triplicate measurements demonstrated that the assay could reliably detect EVA71 RNA down to a concentration of 3.36 × 10^0^ copies/μL (Figure 4). Consistently strong fluorescent signals were observed for samples containing EVA71 RNA at levels ranging from 3.36 × 10^7^ to 3.36 × 10^0^ copies/μL, whereas no detectable fluorescence was observed at 3.36 × 10^−1^ copies/μL or in the blank control. These results indicate that the limit of detection for this assay is 3.36 copies per reaction.

### 3.5 Specificity of the EVA71-RT-MCDA-CRISPR assay

**Figure 5:**
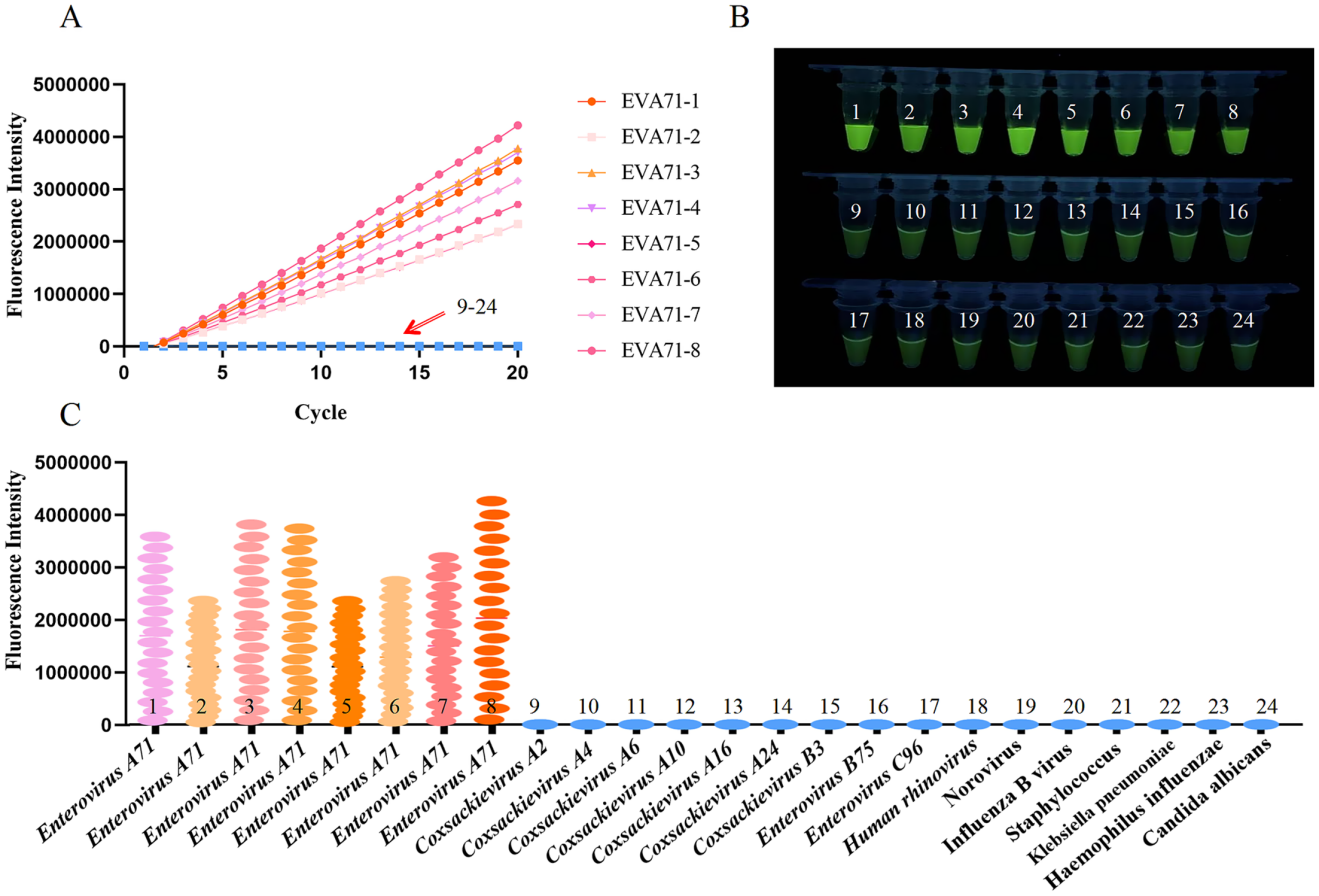
Specificity evaluation of the EVA71-RT-MCDA-CRISPR assay. **(A)** All eight EVA71 strains exhibited positive reaction curves, while no amplification was detected for non-EVA71 pathogens. **(B)** The fluorescence signals were monitored and analyzed under blue-light illumination. **(C)** The fluorescence intensity of 8 EVA71 strains and 16 non-EVA71 pathogens. 1–8: EVA71 strains; 9–24: non-EVA71 pathogens.

The diagnostic specificity of the EVA71-RT-MCDA-CRISPR method was evaluated by testing eight EVA71 isolates together with 16 non-EVA71 pathogenic microorganisms (Supplementary Table S1). Positive fluorescence signals were observed only in samples containing EVA71 strains, whereas no detectable fluorescence was generated from any of the non-EVA71 pathogens (Figure 5). Collectively, these results demonstrate that the proposed method exhibits 100% specificity for EVA71, with no cross-reactivity against non-EVA71 pathogens.

### 3.6 Operator variability and reproducibility of the EVA71-RT-MCDA-CRISPR assay

operator variability: Assays were conducted independently by two operators with their own prepared reagents. CV was calculated as 5.52% at low concentration and 3.25% at high concentration, verifying reliable consistency among different operators. Intra-batch and inter-batch variation: We detected low, medium, and high concentration EVA71 RNA with three replicates per concentration. The intra-batch coefficient of variation (CV) was 3.19–3.96%, and the inter-batch CV was 5.31–6.40%, indicating excellent repeatability (Supplementary Table S2).

### 3.7 Clinical feasibility verification of the EVA71-RT-MCDA-CRISPR detection system

To evaluate the diagnostic performance of the EVA71-RT-MCDA-CRISPR technique, parallel testing was performed on 96 clinical specimens (44 anal swabs and 52 throat swabs) obtained from individuals suspected of EVA71 infection. The proposed assay identified 22 positive samples and 74 negative samples, and all results were subsequently confirmed by RT-qPCR analysis (Supplementary Table S3).

**Figure 6:**
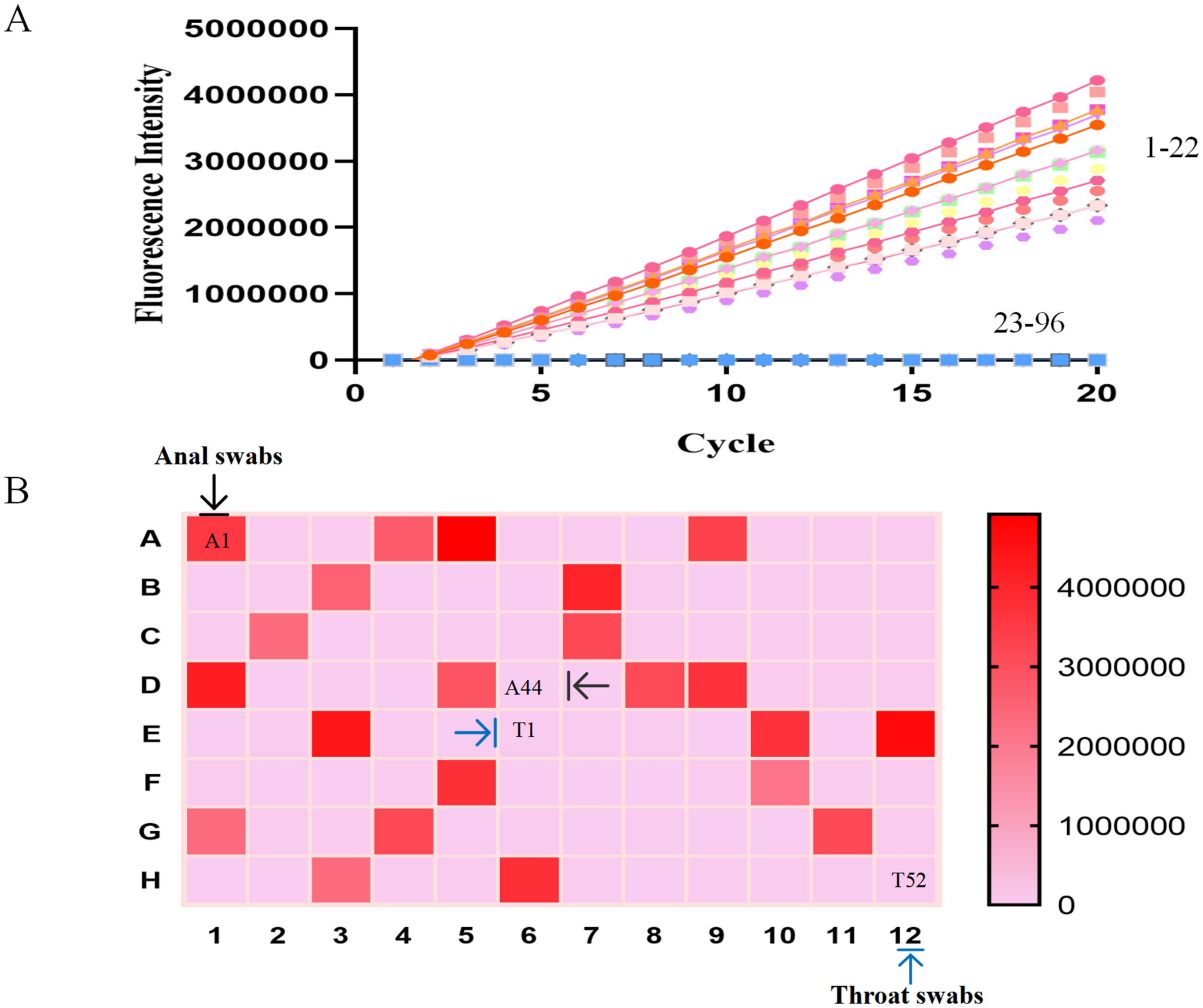
Application of the EVA71-RT-MCDA-CRISPR assay in clinical samples. **(A)** EVA71-RT-MCDA-CRISPR assay. 1–22: the EVA71 positive samples, 23–96: the EVA71 negative samples. **(B)** Heatmap generated based on fluorescence endpoint values using the EVA71-RT-MCDA-CRISPR assay. A1–A44: anal swabs, T1–T52: throat swabs.

Compared with traditional qRT-PCR technique, our assay’s sensitivity and specificity were 100% (95% CI, 84.56–100%) and 100% (95% CI, 95.14–100.00%), respectively (Figure 6 and Supplementary Table S4). This comparative validation demonstrates that the EVA71-RT-MCDA-CRISPR approach offers accurate and reliable detection of EVA71 in clinical specimens.

## 4 Discussion

*Enteroviruses* are highly contagious and exhibit a strong affinity for neural tissues (Tian et al., 2025). Research indicates that the neurotropic characteristics of EVA71 and elevated viral loads are directly associated with disease severity, while the pathogen also demonstrates considerable genetic instability (Wenwen et al., 2023). At present, no targeted therapeutic agents are available for the treatment of HFMD (Guan et al., 2017). Although China’s NMPA has approved inactivated EVA71 vaccine that depicts substantial efficacy against EVA71-induced HFMD, the co-circulation of diverse pathogenic variants and evolving microbial epidemiology limits the effectiveness of single-pathogen countermeasures (Chen et al., 2023; Hu et al., 2024). Consequently, developing advanced EVA71 diagnostic methods is essential for improving detection accuracy and outbreak surveillance (Hu et al., 2024). In this study, we established an innovative EVA71 detection system, termed EVA71-RT-MCDA-CRISPR, which integrates MCDA amplification technology with the CRISPR/Cas12a detection platform. The method enables rapid and accurate detection of EVA71 in clinical samples through advanced technological approaches.

Currently, CRISPR/Cas systems have become state-of-the-art platforms for nucleic acid detection and hold considerable promise for molecular diagnostic applications (Jia et al., 2025a). The discovery of CRISPR-based molecular tools and their associated catalytic components—including Cas9, Cas12, Cas13, and Cas14—has greatly advanced POCT by enabling precise, portable, and highly sensitive pathogen detection (Phan et al., 2022). Among these, Cas12a is an RNA-guided DNA-targeting enzyme that has become instrumental for detecting numerous infectious agents (Pickar-Oliver and Gersbach, 2019). Upon recognition and binding to a target DNA sequence adjacent to a PAM site, this protein complex triggers non-specific cleavage of single-stranded DNA probes labeled with fluorophore-quencher pairs (Jia et al., 2023). This cleavage separates the fluorescent reporter from its quencher, producing a measurable fluorescence signal that can be quantified using standard fluorescence detection equipment (Gootenberg et al., 2017). The versatility of this system can be further enhanced by customizing the guide RNA sequence, making it particularly attractive for nucleic acid-based pathogen screening applications (Liu et al., 2023).

The integration of CRISPR/Cas systems with isothermal amplification technologies can effectively improve the identification accuracy of target genes and broaden the detection scope (Wang S. et al., 2024, Wang X. et al., 2024). Such as recombinase polymerase amplification (RPA) and loop-mediated isothermal amplification (LAMP) have been integrated with CRISPR-based detection to develop molecular diagnostic techniques represented by Specific High-Sensitivity Enzymatic Reporter UnLOCKing (SHERLOCK)(Ali et al., 2020; Gootenberg et al., 2017; Li et al., 2022; Shen et al., 2023). Nevertheless, only a small number of studies have adopted detection strategies integrating isothermal amplification and CRISPR technology for the identification of HFMD-related pathogens (Lin et al., 2023). This indicates that further in-depth research is still needed in this field. Currently, the prevailing isothermal amplification techniques for such assays are RPA and LAMP (Lin et al., 2023; Zhuang et al., 2025). Notably, no study has yet integrated the MCDA technique with the CRISPR/Cas system for HFMD detection. Accordingly, this study innovatively combines the isothermal amplification technique MCDA with the CRISPR/Cas12a system to establish a new detection method for HFMD viruses. Compared with RPA and LAMP (Supplementary Table S5), MCDA represents an emerging isothermal amplification technique (Wang et al., 2015). It exhibits remarkable economic advantages over RPA, as only strand-displacement DNA polymerase is required for amplification (Tan et al., 2022; Zhu et al., 2021). Whereas LAMP typically uses 4–6 primers, MCDA can employ up to 10 primers to achieve higher detection specificity and reduce false-positive results (Wang et al., 2015, 2024).

The sensitivity of the EVA71-RT-MCDA-CRISPR assay demonstrated that this assay could detect EVA71 RNA templates at 3.36 copies per reaction. It also had excellent specificity, no cross-reactivity with non-EVA71 pathogens. In the clinical feasibility assessment, this assay demonstrated satisfactory diagnostic efficacy for EVA71 infection, accurately identifying 22 positive specimens and 74 negative specimens. The detection results were consistent with those obtained via RT-qPCR. The entire detection process could be completed within 70 min, which included nucleic acid extraction (15 min), RT-MCDA reaction (40 min), and CRISPR-based cleavage (15 min).

We conducted a systematic comparison of the EVA71-RT-MCDA-CRISPR assay with three isothermal amplification-CRISPR/Cas platforms for the detection of HFMD viruses (Supplementary Table S6): Compared with Marples (Cas12a-based RPA multiplex assay, LOD: 1 copy/μL)(Lin et al., 2023) and HICAS-Chip (a heparin-assisted one-pot RT-RPA/CRISPR-Cas12a microfluidic platform, LoD: 6.0 copies/μL)(Chen et al., 2026), our method has advantages in operation and cost. The cost per reaction was approximately $2.87 (Supplementary Table S7). Compared with the HNB-RT-LAMP–CRISPR/Cas12a assay (a one-tube platform that integrates hydroxynaphthol blue-based RT-LAMP with CRISPR/Cas12a, LoD: 1–10 copies/μL)(Zhuang et al., 2025), our EVA71-RT-MCDA-CRISPR system features a simpler primer design and a significantly lower risk of non-specific amplification. In our experimental design, a TTTC PAM sequence was integrated into the linker region of primer CP1. Although the original target region lacks a native PAM site that would normally facilitate CRISPR/Cas12 recognition, our system still generates substantial amounts of amplified DNA fragments containing PAM sequences. This feature enables the EVA71-RT-MCDA-CRISPR detection method to function effectively without requiring pre-existing PAM sites in the target DNA (Ng et al., 2025).

As stated above, the EVA71-RT-MCDA-CRISPR assay features rapidity, high sensitivity and excellent specificity. The visual observation protocol of the EVA71-RT-MCDA-CRISPR platform can be achieved using conventional basic thermostatic equipment or even a simple constant-temperature water bath. The reaction system requires two constant temperatures of 65 °C and 37 °C, together with a blue light illuminator for result observation. This method shows great potential for the clinical diagnosis of EVA71 infection in POCT and resource-limited regions. Meanwhile, it lays a foundation for the multiplex detection of HFMD-associated pathogens based on the MCDA-CRISPR platform, and also provides a practical and flexible strategy for the identification of other pathogenic microorganisms.

Nevertheless, this detection method still presents certain limitations. The commercial nucleic acid extraction kits used in this study depend on specialized laboratory equipment for sample processing. Furthermore, the necessity of opening the reaction tube during the experimental workflow introduces a risk of aerosol contamination, which represents a notable drawback of the EVA71-MCDA-CRISPR system due to temperature incompatibility between the MCDA and CRISPR/Cas12a components. Therefore, future research will prioritize physical separation techniques (Zhuang et al., 2025), nanobiosensors (Phan et al., 2022) and microfluidic platforms (Shen et al., 2023; Xie et al., 2025). The above technologies can complete all reaction procedures via closed-tube or one-pot strategies, fundamentally reducing the hidden danger of aerosol contamination. These approaches will be the primary research directions for our subsequent assay optimization.

Furthermore, we solely validated the assay using 96 anal and throat swabs, which represents a limited sample size. A larger number of samples encompassing a wider range of specimen types, such as stool and blood, is required to further evaluate its extensive clinical applicability. Meanwhile, absorbance thresholds can be set for negative controls in visual detection to eliminate subjective bias from naked-eye judgment, promoting the application of isothermal amplification technology in POCT. Thereby enhancing the method’s potential utility in public health surveillance and epidemic response.

## 5 Conclusion

In conclusion, this study successfully designed and established an EVA71-RT-MCDA-CRISPR detection method for EVA71 by integrating RT-MCDA with CRISPR/Cas12a technology. The EVA71-RT-MCDA-CRISPR technique exhibits outstanding analytical performance, attaining a detection limit of 3.36 copies per reaction within 70 min. This platform demonstrated excellent performance in the analysis of clinical samples, accurately identifying EVA71 infection without cross-reactivity against non-EVA71 pathogens. Furthermore, this approach has been proven to possess high sensitivity and specificity in clinical settings. Therefore, the newly developed EVA71-RT-MCDA-CRISPR assay represents a promising tool for the diagnosis of EVA71 infection. This study also provides a foundation for the establishment of similar detection assays for other infectious pathogens.

## Data Availability

The datasets presented in this study can be found in online repositories. The names of the repository/repositories and accession number(s) can be found in the article/Supplementary material.
