# Supplementary material for: A CRISPR/Cas12a-based reverse transcription multiple cross displacement amplification technique for sensitive, rapid, and highly specific detection of human enterovirus A71 in clinical application
Source: Front Microbiol. 2026 Jun 19;17:1883920. doi: 10.3389/fmicb.2026.1883920 (PMC13328427; doi:10.3389/fmicb.2026.1883920)
Supplement: Supplementary file 1 [file Supplementary_file_1.DOCX]

**A CRISPR/Cas12a-based Reverse Transcription** **Multiple Cross Displacement Amplification Technique for sensitive, Rapid, and Highly Specific Detection of *Human Enterovirus A71* in Clinical Application**

***Xujian Zhang^1,2^, Rui Ye^2,3^, Qi Liang^3,4^, Yumei Cao^3,4^, Mao Liu^3,4^, Fang Wei^2^, Yonglin Zhu^2^, Yan Yuan^2^, Guo Guo^1*^, Yu Wang^2,3*^***

*^1^ School of Basic Medical Sciences, Guizhou Key Laboratory of Microbio and Infectious Disease Prevention & Control, Guizhou Medical University, Guiyang, Guizhou, China.*

*^2^ Department of Clinical Laboratory, The First People's Hospital of Guiyang, Guiyang, Guizhou, China.*

*^3^ Department of Basic Clinical Laboratory Medicine, School of Clinical Laboratory Science, Guizhou Medical University, Guiyang, Guizhou, China.*

*^4^ School of Public Health, Key Laboratory of Environmental Pollution Monitoring and Disease Control, Ministry of Education, Guizhou Medical University, Guiyang, Guizhou, China.*

****Correspondence: Yu Wang:wangzhongyuwy@163.com.***

****Correspondence: GuoGuo:guoguojsc@163.com.***

**Inventory of Supplementary Information:**

1. **Supplementary Figures**

Figure S1 Primers for the VP1 gene used in the EVA71-RT-MCDA-CRISPR assay in this study.

Figure S2 Optimal amplification temperature for EVA71-RT-MCDA assay.

Figure S3 Temperature optimisation for EVA71-RT-MCDA-CRISPR assay.

Figure S4 Time optimisation for EVA71-RT-MCDA-CRISPR assay.

1. **Supplementary tables**

Table S1 Pathogens used in the current study.

Table S2 Operator variability and reproducibility of the EVA71-RT-MCDA-CRISPR assay.

Table S3 Clinically information of the child who participate this study.

Table S4 Comparison of RT‐qPCR and EVA71-RT-MCDA-CRISPR methods to identify EVA71 in clinical samples.

Table S5 Comparison of MCDA and other common isothermal amplification methods.

Table S6 Performance comparison of the EVA71-RT-MCDA-CRISPR assay with other recently reported isothermal amplification methods for enterovirus detection.

Table S7 Presents a summary of the cost per reaction associated with the EVA71-RT-MCDA- CRISPR assay.

**Figures**

**Figure S1. Primers for the VP1 gene used in the EVA71-RT-MCDA-CRISPR assay in this study.**

Location and sequences of primers and gRNA used in this study. The MCDA primers are positioned with arrows, and the gRNA is in the box. Right and left arrows indicate the sense and complementary sequences used, respectively.

**
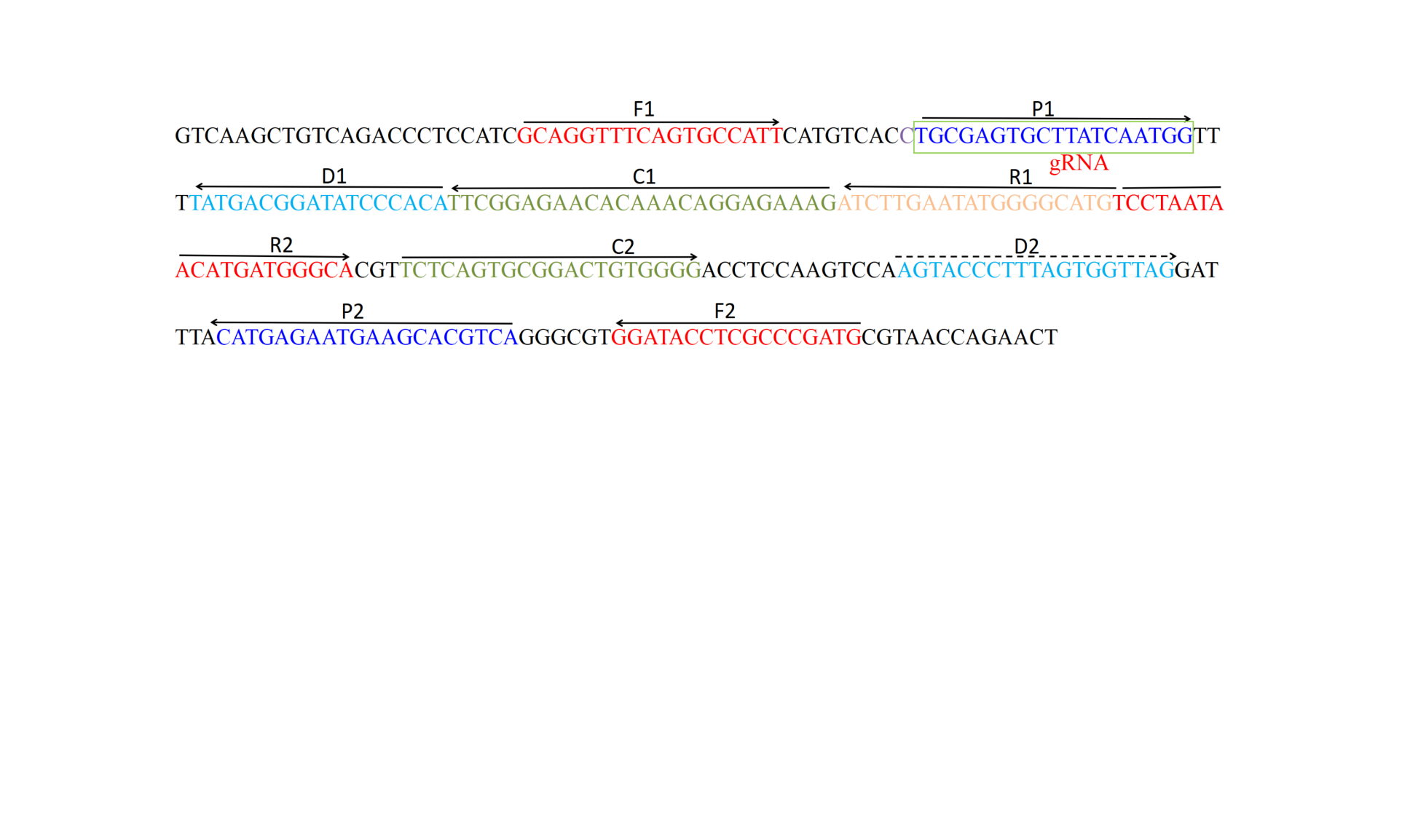
**

**Figure S2 Optimal amplification temperature for EVA71-RT-MCDA assay.**

By using a real‐time measurement to monitor the turbidity of EVA71‐RT‐MCDA reactions. (A-H) Eight kinetic curves were generated from 61 to 68 ℃ (1 ℃ intervals), At 65 ℃, the reaction system reached the absorbance threshold of 0.1 (the criterion for positive amplification) most rapidly, and thus 65 ℃ was determined as the optimal temperature for EVA71‑RT‑MCDA amplification.

**
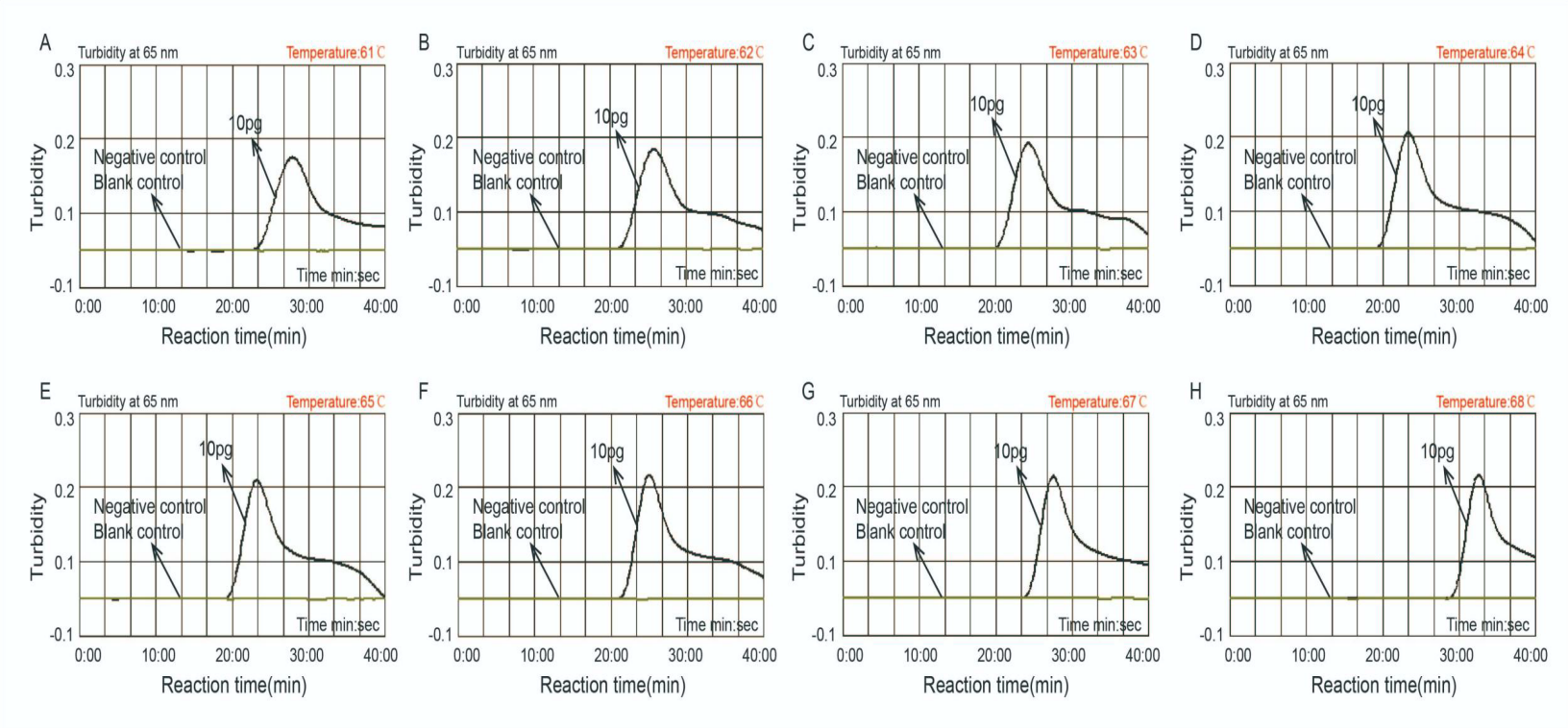
**

**Figure S3 Temperature optimisation for EVA71-RT-MCDA-CRISPR assay.**

The optimal temperature for the EVA71-RT-MCDA-CRISPR assay was evaluated by comparing CRISPR/Cas12a step reactions performed at six constant temperatures ranging from 35 to 40 °C. The reaction process was monitored by fluorescence signal detection and blue-light illumination. (A-F) The real time fluorescence assay. (H-I) respectively represent the differences in fluorescence intensity at temperatures from 35-40℃. (J) The blue-light illumination using ImageJ software.

**
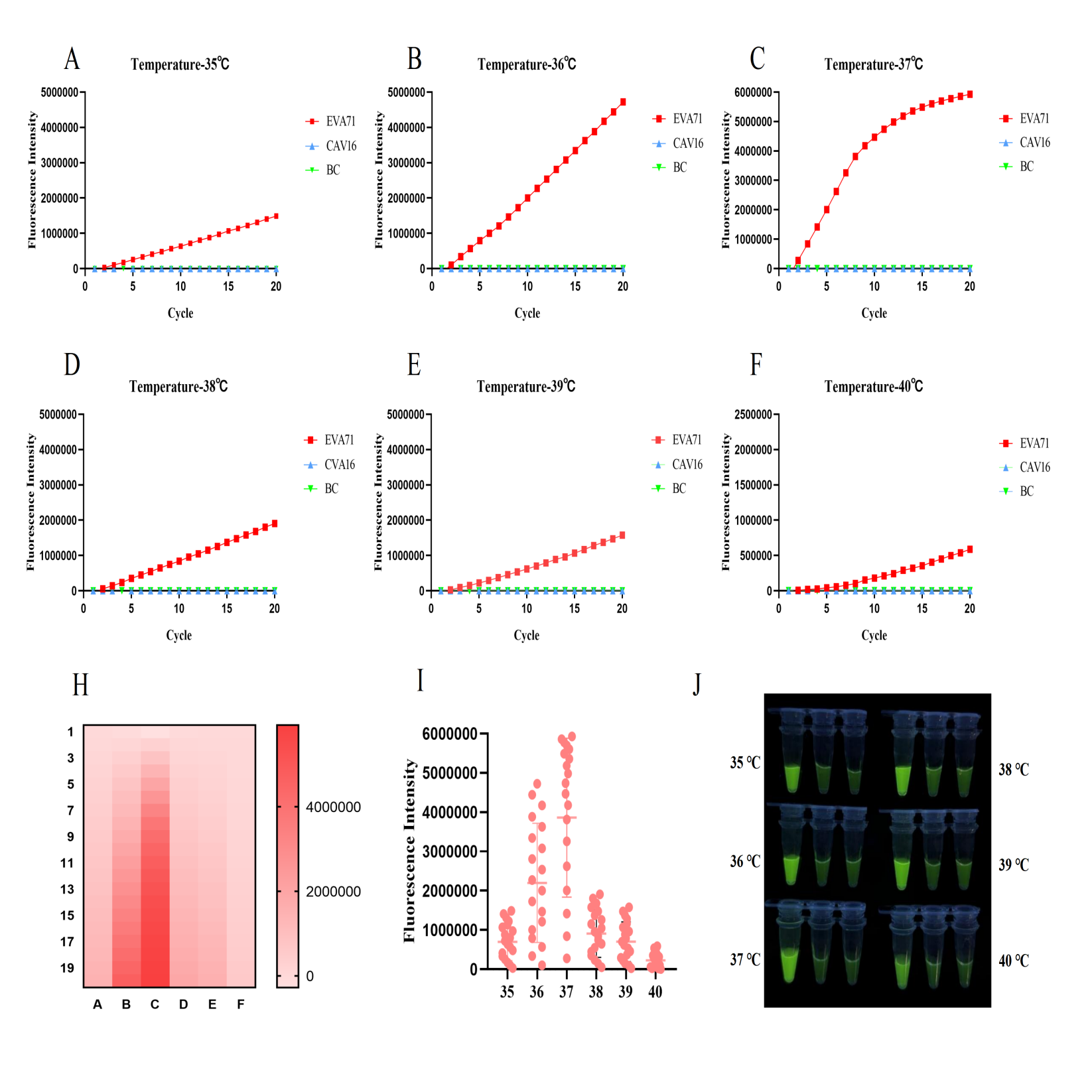
**

**Figure S4 Time optimisation for EVA71-RT-MCDA-CRISPR assay.**

To optimize the reaction time for CRISPR/Cas12a trans-cleavage, intervals of 5, 10, 15, and 20 min were tested at 37 ℃, with 5 min found to be sufficient for effective cleavage of the ssDNA reporter. while the maximum fluorescence signal was observed in 15 min. The reaction process was monitored by fluorescence signal detection and blue-light illumination. (A-D) The real time fluorescence assay. (E) represent the differences in fluorescence intensity at 5 to 20 minutes. (F) The blue-light illumination.


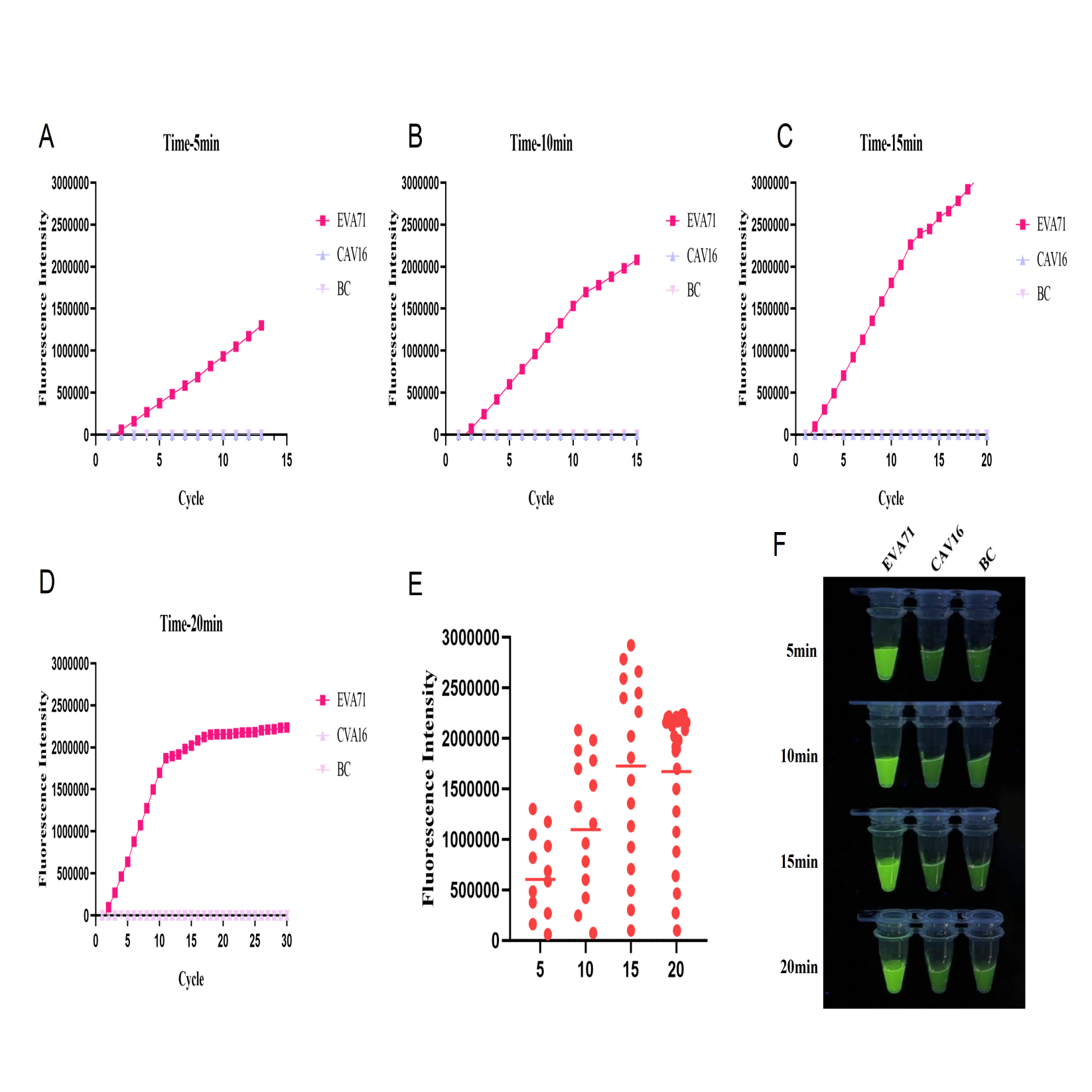


**Tables**

**Table S1 Pathogens used in the current study.**

| **Pathogen** | **Source of Pathogens^a^** | **No.of strains** | **EVA71-RT-MCDA-CRISPR Result*^b^*** |
| --- | --- | --- | --- |
| *Enterovirus A71* (EVA71) | Isolated strains(GZCDC) | 8 | P |
| *Coxsackievirus A2* (CVA2) | Isolated strains(GZCDC) | 1 | N |
| *Coxsackievirus A4* (CVA4) | Isolated strains(GZCDC) | 1 | N |
| *Coxsackievirus A6* (CVA6) | Isolated strains(GZCDC) | 1 | N |
| *Coxsackievirus A10*(CVA16) | Isolated strains(GZCDC) | 1 | N |
| *Coxsackievirus A16* (CVA10) | Isolated strains(GZCDC) | 1 | N |
| *Coxsackievirus A24* (CVA24) | Isolated strains(GZCDC) | 1 | N |
| *Coxsackievirus B3* (CVB3) | Isolated strains(GZCDC) | 1 | N |
| *Enterovirus B75* (EVB75) | Isolated strains(GZCDC) | 1 | N |
| *Enterovirus C96* (EVC96) | Isolated strains(GZCDC) | 1 | N |
| *Human rhinovirus (clinical samples)* | GFPH | 1 | N |
| *Norovirus (clinical samples)* | GFPH | 1 | N |
| *Influenza B virus (clinical samples)* | GFPH | 1 | N |
| *Staphylococcus aureus* | Isolated strains(GFPH) | 1 | N |
| *Klebsiella pneumoniae* | Isolated strains(GFPH) | 1 | N |
| *Haemophilus influenzae* | Isolated strains(GFPH) | 1 | N |
| *Candida albicans* | ATCC10231 | 1 | N |

*^a^*GZCDC*,Guizhou Provincial Center for Disease Control and Prevention;* GFPH*:The First People’s Hospital of Guiyang；*^b^P, positive; N, negative.

**Table S2 Operator variability and reproducibility of the EVA71-RT-MCDA-CRISPR assay.**

| Pathogen | Concentrations of template | Coefficient of variation (CV) | | |
| --- | --- | --- | --- | --- |
|  |  | Operational variability | Intra-batch | Inter-batch |
| EVA71 | 3.36×10^0^ copies/μL | 5.52% | 3.96% | 6.40% |
|  | 3.36×10^3^ copies/μL | / | 3.19% | 5.52% |
|  | 3.36×10^6^ copies/μL | 3.25% | 3.21% | 5.31% |

**Table S3 Clinically information of the child who participate this study.**

| Sample NO. | Sample types | Sex^a^ | Age^b^ | EVA71-RT-qPCR | | EVA71-RT-MCDA-CRISPR^e^ |
| --- | --- | --- | --- | --- | --- | --- |
|  |  |  |  | P/N^c^ | CT Value^d^ |  |
| 1 | Anal swabs | M | 5 | P | 20.23 | P |
| 2 | Anal swabs | F | 12 | N | - | N |
| 3 | Anal swabs | F | 0 | N | - | N |
| 4 | Anal swabs | F | 2 | P | 15.10 | P |
| 5 | Anal swabs | M | 5 | N | - | N |
| 6 | Anal swabs | M | 8 | N | - | N |
| 7 | Anal swabs | F | 12 | P | 28.50 | P |
| 8 | Anal swabs | M | 1 | N | - | N |
| 9 | Anal swabs | F | 3 | N | - | N |
| 10 | Anal swabs | F | 4 | N | - | N |
| 11 | Anal swabs | M | 12 | P | 27.21 | P |
| 12 | Anal swabs | M | 10 | N | - | N |
| 13 | Anal swabs | M | 1 | N | - | N |
| 14 | Anal swabs | F | 3 | N | - | N |
| 15 | Anal swabs | F | 2 | N | - | N |
| 16 | Anal swabs | M | 7 | N | - | N |
| 17 | Anal swabs | F | 5 | N | - | N |
| 18 | Anal swabs | M | 8 | P | 27.46 | P |
| 19 | Anal swabs | M | 7 | N | - | N |
| 20 | Anal swabs | F | 3 | N | - | N |
| 21 | Anal swabs | M | 6 | P | 18.97 | P |
| 22 | Anal swabs | F | 4 | N | - | N |
| 23 | Anal swabs | F | 3 | N | - | N |
| 24 | Anal swabs | M | 4 | P | 22.36 | P |
| 25 | Anal swabs | M | 5 | P | 24.17 | P |
| 26 | Anal swabs | M | 3 | N | - | N |
| 27 | Anal swabs | F | 6 | N | - | N |
| 28 | Anal swabs | F | 7 | N | - | N |
| 29 | Anal swabs | F | 3 | N | - | N |
| 30 | Anal swabs | M | 8 | N | - | N |
| 31 | Anal swabs | F | 6 | P | 25.32 | P |
| 32 | Anal swabs | M | 7 | N | - | N |
| 33 | Anal swabs | M | 6 | P | 15.14 | P |
| 34 | Anal swabs | F | 8 | N | - | N |
| 35 | Anal swabs | M | 5 | N | - | N |
| 36 | Anal swabs | F | 4 | P | 21.25 | P |
| 37 | Anal swabs | F | 5 | N | - | N |
| 38 | Anal swabs | F | 7 | P | 19.98 | P |
| 39 | Anal swabs | M | 8 | N | - | N |
| 40 | Anal swabs | M | 2 | N | - | N |
| 41 | Anal swabs | F | 3 | N | - | N |
| 42 | Anal swabs | F | 5 | N | - | N |
| 43 | Anal swabs | M | 1 | N | - | N |
| 44 | Anal swabs | F | 5 | N | - | N |
| 45 | Throat swabs | M | 2 | N | - | N |
| 46 | Throat swabs | M | 6 | N | - | N |
| 47 | Throat swabs | M | 5 | N | - | N |
| 48 | Throat swabs | F | 8 | P | 21.42 | P |
| 49 | Throat swabs | F | 3 | N | - | N |
| 50 | Throat swabs | F | 5 | P | 23.76 | P |
| 51 | Throat swabs | F | 4 | P | 25.88 | P |
| 52 | Throat swabs | M | 2 | N | - | N |
| 53 | Throat swabs | M | 1 | N | - | N |
| 54 | Throat swabs | F | 7 | N | - | N |
| 55 | Throat swabs | M | 11 | N | - | N |
| 56 | Throat swabs | F | 13 | N | - | N |
| 57 | Throat swabs | F | 9 | N | - | N |
| 58 | Throat swabs | M | 6 | N | - | N |
| 59 | Throat swabs | M | 5 | N | - | N |
| 60 | Throat swabs | F | 7 | P | 24.65 | P |
| 61 | Throat swabs | M | 8 | N | - | N |
| 62 | Throat swabs | F | 4 | N | - | N |
| 63 | Throat swabs | F | 3 | N | - | N |
| 64 | Throat swabs | M | 3 | N | - | N |
| 65 | Throat swabs | M | 2 | P | 22.15 | P |
| 66 | Throat swabs | F | 3 | N | - | N |
| 67 | Throat swabs | M | 5 | N | - | N |
| 68 | Throat swabs | M | 6 | P | 23.36 | P |
| 69 | Throat swabs | M | 6 | N | - | N |
| 70 | Throat swabs | M | 7 | N | - | N |
| 71 | Throat swabs | F | 5 | N | - | N |
| 72 | Throat swabs | F | 7 | N | - | N |
| 73 | Throat swabs | M | 8 | N | - | N |
| 74 | Throat swabs | F | 4 | N | - | N |
| 75 | Throat swabs | F | 5 | N | - | N |
| 76 | Throat swabs | M | 2 | N | - | N |
| 77 | Throat swabs | F | 9 | P | 18.90 | P |
| 78 | Throat swabs | F | 2 | P | 27.80 | P |
| 79 | Throat swabs | F | 10 | N | - | N |
| 80 | Throat swabs | M | 11 | N | - | N |
| 81 | Throat swabs | M | 2 | N | - | N |
| 82 | Throat swabs | F | 5 | N | - | N |
| 83 | Throat swabs | M | 8 | N | - | N |
| 84 | Throat swabs | F | 5 | N | - | N |
| 85 | Throat swabs | M | 2 | N | - | N |
| 86 | Throat swabs | M | 11 | N | - | N |
| 87 | Throat swabs | M | 9 | P | 22.47 | P |
| 88 | Throat swabs | M | 7 | N | - | N |
| 89 | Throat swabs | F | 4 | N | - | N |
| 90 | Throat swabs | F | 8 | N | - | N |
| 91 | Throat swabs | M | 4 | N | - | N |
| 92 | Throat swabs | F | 6 | N | - | N |
| 93 | Throat swabs | F | 7 | P | 19.60 | P |
| 94 | Throat swabs | M | 3 | N | - | N |
| 95 | Throat swabs | M | 6 | N | - | N |
| 96 | Throat swabs | F | 8 | N | - | N |

^a^F, Female; M, Male；^b^Age (years), The age of 0 was defined as children less than 1 year old；^c/d^P, positive; N, negative.

^d^**­**, represents undefined; CT, represents cycle threshold; ^e^P, positive; N, negative.

**Table S4 Comparison of RT‐qPCR and EVA71-RT-MCDA-CRISPR methods to identify EVA71 in clinical samples.**

| **EVA71-RT-MCDA-CRISPR assay** | **EVA71-RT-qPCR assay**  **(reference method)** | | **Total** | **Sensitivity(%)** | | **Specificity(%)** | |
| --- | --- | --- | --- | --- | --- | --- | --- |
|  | **Positive** | **Negative** |  | **Value** | **95%CI** | **Value** | **95%CI** |
| Positive | 22 | 0 | 22 | 100.00% | 84.56% to 100.00% | 100.00% | 95.14% to 100.00% |
| Negative | 0 | 74 | 74 |  |  |  |  |
| Total | 22 | 74 | 96 |  |  |  |  |

Abbreviations: EVA71, *Enterovirus A71*; MCDA, multiple cross displacement amplification; CRISPR,clustered regularly interspaced short palindromic repeat; RT‐qPCR, reverse transcription polymerase chain reaction.

**Table S5 Comparison of MCDA and other common isothermal amplification methods.**

| Method | MCDA | LAMP | RPA |
| --- | --- | --- | --- |
| Origin | 2015 | 2000 | 2006 |
| detection time | 40 min | 30 min | 25–40 min |
| Number of primers | 10 | 4-6 | 2 |
| temperatures | 60-65°C | 60-65°C | 37-42°C |
| Detection cost | Low, single‑enzyme system | Low, single‑enzyme system | High, expensive multi‑enzyme complex |
| Advantages | High sensitivity & specificity, low cost, low contamination risk, compatible with CRISPR, suitable for field POCT | Rapid amplification, low cost, well‑established technique | Mild reaction temperature, no heating required, fast amplification |
| Disadvantages | Relatively complex primer design, requires 65 °C heater | Complicated primer design, high non‑specific amplification and false‑positive risk | High reagent cost, vulnerable to environmental interference |

**Table S6 Performance comparison of the EVA71-RT-MCDA-CRISPR assay with other recently reported isothermal amplification methods for enterovirus detection.**

| Method | detection scope (target virus) | limit of detection (LoD) | detection time(Including extraction) | workflow complexity | equipment needed | sample types validated | cost (estimated) |
| --- | --- | --- | --- | --- | --- | --- | --- |
| EVA71-RT-MCDA-CRISPR  **(this study)** | EVA71 | 3.36 copies  per reaction | 70 min | Medium | 37 ℃ and 65 ℃  constant-temperature device  blue light illuminator or fluorescence microplate reader | 44 anal swabs  52 throat swabs | Low-Medium |
| MARPLES (RPA-CRISPR/Cas12a) | Multiplex detection | 1.0 copies/μL | 60 min | High | 37℃ constant-temperature device  UV torch or a multifunctional  microplate reader | 56 clinical  specimens | High |
| HICAS-Chip (Microfluidic RT-RPA-CRISPR/Cas12a) | Multiplex detection | 10 aM  (6.0 copies/μL) | 60 min | High | String-powered microfluidic chip, heating pad | 24 Wastewater samples | High |
| HNB-RT-LAMP–CRISPR/Cas12a | both EVs and EV-A71 | EVA71:1.0 copies/μL  EVs:10 copies/μL | 65 min | Medium | 37 ℃ and 65 ℃  constant-temperature device  natural /blue light transilluminator | 94 NP swab samples | Low-Medium |

**Table S7 Presents a summary of the cost per reaction associated with the EVA71-RT-MCDA -CRISPR assay.**

|  | Reagent | Specification | Total Price(¥) | Single Dosage | Cost per Sample(¥) | Cost per Sample($) |
| --- | --- | --- | --- | --- | --- | --- |
| RNA extraction | nucleic acid extraction kits | 50 T | 320 | / | 6.4 | 0.95 |
| RT-MCDA pre-amplification reaction | 10× isothermal buffer II | 6 mL (4×1.5 mL) | 499 | 2.5 μL | 0.21 | 0.03 |
|  | MgSO4 | 6 mL (4×1.5 mL) | 409 | 1.5 μL | 0.14 | 0.02 |
|  | dNTP | 5×0.8 mL | 2,998 | 3.5 μL | 2.62 | 0.39 |
|  | Bst 3.0 | 1 mL | 3,100 | 1 μL | 3.1 | 0.46 |
|  | AMV reverse transcriptase | 1000μL | 1100 | 1 μL | 1.1 | 0.16 |
|  | RNase-free Water | 1 mLx10 | 67 | 8-12 μL | 0.09 | 0.01 |
| CRISPR/Cas12a trans-cleavage assay | EnGen Lba Cas12a | 100 uM(20 μL) | 2,742 | 10 μL(1 uM) | 0.14 | 0.02 |
|  | 10× NEBuffer | 5 mL | 359 | 25 μL | 0.42 | 0.06 |
|  | RNase-free Water | 1 mLx10 | 67 | 9-13 μL | 0.09 | 0.01 |
|  | probe | 350 μL | 1500 | 1.2 μL | 5 | 0.74 |
|  | gRNA | 100 uM (20 μL) | 2200 | 10 μL(1 uM) | 0.11 | 0.02 |
| Total |  | | | | ¥19.42 | $2.87 |

Abbreviations: ¥, Chinese Yuan (CNY); $, US Dollar (USD).
